# The Effects of Oral Nicotine Pouch Packaging Features on Adult Tobacco Users’ and Non-Users’ Product Perceptions

**DOI:** 10.3390/ijerph20043383

**Published:** 2023-02-15

**Authors:** Darren Mays, Lauren Long, Mahmood A. Alalwan, Theodore L. Wagener, Ce Shang, Megan E. Roberts, Joanne G. Patterson, Brittney Keller-Hamilton

**Affiliations:** 1 Department of Internal Medicine, The Ohio State University College of Medicine, Center for Tobacco Research, The Ohio State University Comprehensive Cancer Center, Columbus, OH 43214, USA; 2 Division of Epidemiology, College of Public Health, The Ohio State University, Columbus, OH 43210, USA; 3 Division of Health Behavior and Health Promotion, College of Public Health, The Ohio State University, Columbus, OH 43210, USA

**Keywords:** packaging, marketing, consumer perceptions, nicotine

## Abstract

Background: Oral nicotine pouches (ONPs) are novel products that are marketed as “tobacco-free” alternatives to cigarettes and smokeless tobacco (ST). This study examined the effects of ONP packaging features on adult tobacco users’ and non-users’ product perceptions. Materials and Methods: Adult tobacco users (cigarettes, ST, and dual cigarette/ST) and non-users (total N = 301) viewed ONP pack images in a 4 × 3 × 2 between-subject experiment testing the effects of the displayed flavor (cool mint, coffee, dark frost, and smooth), nicotine concentration (none displayed on the package, 3 mg, and 6 mg), and addiction warning label (yes or no). The outcomes were perceived substitutability of ONPs for cigarettes and ST and perceived risks. We modeled the effects of tobacco user status and the experimental factors on these outcomes. Results: All tobacco user groups perceived ONPs to be significantly less harmful and less addictive than non-users. There were significant effects of nicotine concentration on perceived risks. Compared to packages that did not display nicotine concentration, packages displaying 6 mg nicotine concentration produced significantly lower perceived harm (*β* = −0.23, 95% CI −0.44, −0.02), perceived addictiveness (*β* = −0.28, 95% CI −0.51, −0.05), risk appraisals of harm (*β* = −0.50, 95% CI −0.88, −0.12) and risk appraisals of addictiveness (*β* = −0.53, 95% CI −0.95, −0.11). Conclusions: The study findings demonstrate that the nicotine concentration displayed on ONP packaging can affect adults’ perceptions of ONPs. Further research on the effects of ONP packaging features emphasizing nicotine (e.g., “tobacco free” nicotine claims) on tobacco users and non-users is needed to assess their potential public health impact.

## 1. Introduction

Oral nicotine pouches (ONPs), a novel type of nicotine product, have recently emerged in the USA and have been available nationally since 2019 [1,2,3]. ONPs come in packages of pre-portioned pouches containing nicotine, flavorings, fillers, and other ingredients [4,5,6]. ONPs do not contain tobacco leaf, are used between the cheek and gum to deliver nicotine, and do not require spitting as traditional smokeless tobacco does (ST; e.g., chew and dip) [6]. In the last 3 years, the ONP brands available in the USA have proliferated (e.g., Zyn, On!, Rogue, and Velo) and sales data indicate the popularity of ONPs is surging. From 2016 to 2020, ONP sales at U.S. retail stores increased from 0.2 million to nearly 46 million units [3]. Although data on the prevalence of ONP use are limited, recent studies indicate awareness and use of ONPs is highest in adults who use other types of tobacco, especially ST, and that ONPs may appeal to adults who do not use tobacco as well [7,8,9].

Consumers initially encounter novel tobacco products by engaging with marketing, and tobacco manufacturers carefully design marketing to enhance the appeal of tobacco products, affect consumer perceptions, and increase use [10]. Unfettered marketing is described as a “cause of the global spread of tobacco use and addiction” [11,12,13], and manufacturers shifted to packaging as a primary marketing vehicle following policies in the USA and other settings that increasingly restricted tobacco marketing channels (e.g., television) [10,11,14]. Packaging features such as conveying constituents, claims, and descriptors influence the appeal of tobacco products and the likelihood of use [10,11]. Packaging is essential to tobacco marketing (e.g., point-of-sale power walls and advertising), and is used to target specific groups (e.g., young people) [10,11,14]. Policies and regulations targeting packaging include standardized or “plain” packaging, requiring health warnings on tobacco packaging, and prohibiting specific features (e.g., low, light, and mild descriptors; tar/nicotine yields on cigarette packs) [15], and they can reduce tobacco use and its associated morbidity and mortality [16,17,18]. For novel products such as ONPs, studying effects of packaging features can inform policies and regulations to improve public health [1,19,20,21].

ONPs are marketed as alternatives to cigarettes and ST, with advertising emphasizing they are “spit free”, “smoke free” [22], and can be used in settings where other tobacco use is discouraged or prohibited [23]. ONP packaging features sleek designs, attractive colors, appealing flavors, and emphasizes the unique characteristics of ONPs, such as the variety of nicotine “strengths,” or the nicotine concentration per pouch available. As of 2022, all ONP brands featured nicotine concentration on the packaging [24], and ONP advertising emphasizes nicotine concentration as well. For example, the online retailer Nicokick.com indicates: “the [nicotine] strength you pick will depend on a number of factors” including “whether you are a first-timer or you’ve used nicotine for years” [25]. Zyn advertisements display packaging emphasizing that 3 mg ONPs provide “fresh nicotine satisfaction” and 6 mg ONPs deliver “even more nicotine enjoyment” [26]. Some research has examined how characteristics of ONP marketing such as “tobacco free” claims influence perceptions of ONPs [27]. However, evidence remains limited as to how ONP marketing, including packaging, influences perceptions of ONPs. 

The appeal, uptake, and use of ONPs will be impacted by how packaging features affect perceptions of ONPs, including by tobacco users and non-users [28]. Examining how ONP packaging features affect ONP perceptions can provide evidence to guide policy and regulation surrounding ONPs. In the USA, the Family Smoking Prevention and Tobacco Control Act authorized the Food and Drug Administration (FDA) to regulate the marketing, distribution, and sale of cigarettes, smokeless, and roll-your-own tobacco [21]. The 2016 Deeming Rule expanded this regulatory authority to all tobacco products, and 2022 legislation further expanded the FDA’s authority to products using synthetic nicotine [29]. These laws position the FDA to regulate ONP packaging and grant the FDA authority to enact packaging regulations if evidence demonstrates they are appropriate for the protection of public health [21]. Thus, packaging regulations must be guided by research on how potential regulations impact tobacco users and non-users [19,21].

The risks of health harm and addiction of ONPs are not yet known, but evidence (primarily from industry research) suggests that ONPs may pose less harm than cigarettes and ST for adult tobacco users because they do not involve inhaling combusted tobacco smoke and may expose users to fewer harmful chemicals [30,31]. As research on the health risks of ONPs becomes available, it is critical to understand the effects of ONP packaging features on tobacco users’ and non-users’ perceptions of ONPs to inform potential regulations. Given the limited research in this area, the objective of this study was to experimentally examine the effects of prominent ONP packaging features on adult tobacco users’ and non-users’ perceptions of ONPs to inform future research and potential packaging regulations. 

## 2. Materials and Methods

### 2.1. Participants

From April to September 2021, we recruited a convenience sample of cigarette smokers, ST (chew, snuff, dip, and snus) users, and non-users of cigarettes or ST aged ≥21 years who resided in Ohio, USA for a cross-sectional study. We recruited participants using social media advertising, institutional study registries, and word of mouth referrals. Those responding to recruitment advertisements completed a brief online eligibility screening, assessing their age, tobacco use behavior (i.e., cigarette smoking and ST use), and contact information. We sent those meeting eligibility criteria a secure personal web link to complete a self-report online survey. We reviewed the screening and survey data quality using methods recommended for remote screening and data collection (e.g., contact information accuracy and potential fraudulent or duplicate responses) [32]. In total, 810 participants completed screening, 650 met the initial eligibility screening (80.2%), and 301 (46.3% of those eligible) eligible participants satisfied data quality checks and completed procedures. All participants provided informed consent, and those completing procedures received a $20 gift card for their time. The host institution’s institutional review board approved the study procedures. 

For analyses, we created four tobacco user groups based on participants’ reported use of cigarettes and ST (chew, snuff, dip, and snus), with current users defined as those who reported using the product every day or some days [33]. The tobacco user categories included no current use of cigarettes/ST (non-user, n = 78, 24.9%), exclusive cigarette smoking (n = 53, 17.6%), exclusive ST use (n = 121, 40.2%), and dual use of cigarettes and ST (n = 49, 16.3%).

### 2.2. Procedures

Participants completed initial questions (demographics and tobacco/nicotine use), read a brief description of ONPs, and were randomized to view an ONP pack image in a 4 (flavor) ×3 (nicotine concentration) ×2 (addiction warning label) between-subject design. Participants completed outcome assessments after viewing the pack image.

We obtained the ONP pack images from an online search and digitally edited the images to align with the conditions in the experimental design. All images were for Zyn brand ONPs, the most popular ONP brand based on U.S. sales data [2,3]. Because flavor influences uptake and use of tobacco and nicotine products [34,35,36,37], we included 4 flavors reflecting the most popular ONP flavors [2,3] at the time of the study and the manufacturers’ use of unambiguous (Cool Mint and Coffee) and ambiguous (Smooth and Dark Frost) flavor descriptors. The label color reflected the labels used by the manufacturer for each flavor (i.e., Cool Mint was blue, Coffee was brown, Smooth was light gray, Dark Frost was dark gray). For the nicotine concentration, we edited the pack images to not display nicotine concentration or to display a 3 mg or 6 mg nicotine concentration. We included ONP packages with and without addiction warning labels in our design because at the time of the study some ONPs were not regulated by the U.S. FDA, manufacturers varied in the use and content of warnings, and it was important to account experimentally for potential effects of other information about nicotine (i.e., the concentration) appearing on packs with and without warnings conveying potential risks of nicotine use. For the addiction warning label, the pack images displayed the text-only warning required by the U.S. FDA under the 2016 Deeming Rule (“Warning: This product contains nicotine. Nicotine is an addictive chemical.”) [29] or we edited them to display no warning label. Other than the experimentally manipulated features, we edited pack images to be consistent across conditions (e.g., size and resolution). The stimuli are available from the corresponding author.

### 2.3. Measures

Before viewing the pack image, we assessed demographics, cigarette smoking, ST use, and past 30-day use of other tobacco and nicotine products (large cigars, little cigars, cigarillos, electronic cigarettes, and waterpipe/hookah) [33]. After viewing the pack image, we measured outcomes assessing participants’ perceptions of ONPs. Given ONP manufacturers’ efforts to position ONPs as alternatives to cigarettes and ST [22] and to capture the potential appeal of ONPs among tobacco users and non-users, we assessed perceived substitutability of ONPs for cigarettes and ST in all participants with two items asking “Could this product be used as a substitute for cigarettes/traditional smokeless tobacco (chew, snuff, dip) for people who smoke/use smokeless tobacco?” Responses were on a 1 (Definitely Not) to 7 (Definitely Yes) scale. 

Risk perceptions are consistently associated with tobacco use behavior [38,39], and they are affected by tobacco marketing [28]. We used items from prior research to capture participants’ ONP risk perceptions in response to packaging features [40,41,42,43]. We measured participants’ overall perceived harm of ONPs by asking “How harmful do you think this product is to your health?” with response options ranging from 1 (Not at All) to 4 (Very). We used a similar item to capture the overall perceived addictiveness of ONPs with response options from 1 (Not at All Addictive) to 4 (Very Addictive). 

We assessed risk appraisals for health harm and addictiveness using 4 items capturing participants’ perceived likelihood of health harm/addiction and worry about health harm/addiction on a 1 (No Chance/Not at All) to 7 (Certain to Happen/Very Much) scale. We averaged the 2 items assessing risk appraisals for health harm (Cronbach’s α = 0.82) and the 2 items assessing risk appraisals for addiction (Cronbach’s α = 0.70). After participants completed outcome assessments, we measured their awareness of ONPs prior to the study and their lifetime and past 30-day ONP use.

### 2.4. Statistical Analysis

Our recruitment and sample size were informed by a priori power analyses to test the main effects and two-way interactions between the experimental factors (nicotine concentration, flavor, and warning label) and potential covariates. For the main and two-way interaction effects, our sample of ≥300 participants provided 80% power to detect effect sizes as small as *f* = 0.20 with α = 0.05. This is a comparable effect size to prior studies testing the effects of packaging and labeling for ST and other combustible tobacco on similar outcomes [44,45].

For each outcome, we created linear regression models that: (1) included the main effects for tobacco user status and the experimental factors; (2) tested the 2-way interactions between the experimental factors (flavor, nicotine concentration, and warning label); (3) tested the 2-way interactions between the experimental factors and tobacco user status; and (4) for completeness, tested the 3-way interactions between the experimental factors to determine if these needed to be accounted for in our analyses. None of the interactions were statistically significant; so, we report results for the models that included main effects for tobacco user status and the experimental factors. Other sociodemographic and tobacco use characteristics were balanced by randomization; so, we did not adjust for them. The missing data were <0.5% for any given variable; so, we used the complete cases for analyses. We conducted all analyses using R version 4.1.3.

## 3. Results

### 3.1. Participant Characteristics

Table 1 shows the sample characteristics (n = 301). Participants averaged 35.7 (*SD* 11.9) years of age, 77.1% male, 92.0% white race, and 2.3% Hispanic ethnicity, and 53.2% reported less than a college education. Most participants (60.5%) described their overall subjective financial situation as “about average.” Most participants had heard of ONPs before the study (68.1%) and 41.7% had tried ONPs. Overall, 18.6% of participants reported using e-cigarettes in the past month, 11.3% large cigars, 9.3% little cigars, 7.6% cigarillos, and 3.3% waterpipe.

### 3.2. Perceived Substitutability for Cigarettes and Smokeless Tobacco

Outcomes are shown in Table 2. There was one statistically significant effect on participants’ perceived substitutability of ONPs for cigarettes (Table 2). Compared to the pack images that did not display the nicotine concentration, participants who viewed the pack images displaying3 mg nicotine concentration reported a lower perceived substitutability for cigarettes (*β* = −0.49, 95% CI −0.96, −0.02). There were no significant effects on perceived substitutability for ST.

### 3.3. Perceived Harm and Addictiveness

For overall perceived harm, we observed significant effects of tobacco user status and nicotine concentration. Compared with non-users, nearly all tobacco user groups reported significantly lower perceived harm and addictiveness (Table 2). Regarding nicotine concentration, participants viewing the pack images displaying 6 mg nicotine concentration reported significantly lower perceived harm (*β* = −0.23, 95% CI −0.44, −0.02) compared to packs that did not display the nicotine concentration. Participants viewing the pack images displaying 6 mg nicotine concentration also reported significantly lower perceived addictiveness (*β* = −0.28, 95% CI −0.51, −0.05) compared to the pack that did not display the nicotine concentration.

### 3.4. Risk Appraisals for Harm and Addiction 

The outcomes for risk appraisals of health harm and addiction, which capture perceived likelihood and worry about risks, are shown in Table 2. For risk appraisals of health harm, there was a significant effect of tobacco user status, where all tobacco user groups (exclusive cigarette smokers, exclusive ST users, and dual users) reported lower risk appraisals of health harm than non-users (Table 2). For risk appraisals of addiction, there was a similar effect of tobacco user status—all tobacco user groups reported lower addiction risk appraisals than non-users (Table 2). Additionally, there was a significant effect of nicotine concentration, where participants viewing the packs displaying 6 mg nicotine concentration reported lower risk appraisals of harm (*β* = −0.49, 95% CI −0.88, −0.12) and lower addiction risk appraisals (*β* = −0.53, 95% CI −0.95, −0.11) compared to the packs that did not display the nicotine concentration.

## 4. Discussion

This experimental study investigated the effects of ONP packaging features including flavor, presence of an addiction warning label, and nicotine concentration on perceptions of ONPs in a sample of adult tobacco users and non-users. The findings demonstrated that adult tobacco users (cigarette smokers, ST users, and dual cigarette and ST users) perceived ONPs to be less harmful and addictive than non-tobacco users, and that nicotine concentration on the ONP packaging affected perceptions of ONPs. ONPs are a relatively new product, and there is limited research on how ONP marketing, including packaging features, influences consumers. These results add to the nascent research on ONPs and have important implications for future research in this area. 

Our study sample included adults aged 21 years and older who smoked cigarettes, used ST, smoked cigarettes and used ST, or did not use tobacco. For our outcomes that captured perceived risks of health harm and addiction, the tobacco users in our sample perceived ONPs to be lower risk than the non-users. Risk perceptions are consistently associated with tobacco use behavior [38,39], and low perceived risks are likely to be associated with ONP trial and use. These findings align with the limited available data suggesting ONPs are most appealing to adults who currently use other tobacco products, particularly ST [7,8,9]. The patterns we observed may also be influenced by participants’ baseline tobacco use behavior. For adults who did not use tobacco products, ONPs may introduce new risks of health harm and addiction translating to higher perceived risks. For adults who used cigarettes, ST, or both, ONPs may be less risky than their usual products translating to lower perceived risks. The potential health risks of ONP use are not yet known, and as more evidence on their potential health risks becomes available it will be important to continue to examine how tobacco users and non-users perceive the risks of ONPs and how such perceptions relate to ONP use behavior.

We did not observe significant effects of the FDA’s required addiction warning label or the flavor of the ONP products displayed. Regarding the warning label, this is consistent with other evidence that text only warnings have minimal effect on outcomes such as those that we measured [46]. The limited effect may also be due to the single brief exposure in the study. Regarding ONP flavor, although flavors are an important factor contributing to the uptake and use of tobacco products [35,37,47,48], the use of fruit and sweet flavored products is more common in youth and young adults than older adults [47,48]. The lack of a significant effect of flavor could be because we focused on adults versus youth, the flavors we used in our design did not align with prominent flavor preferences (e.g., Spearmint and Wintergreen are the most popular ST flavors [2]), or other factors. It is important in future studies to investigate different strategies for communicating the risks of ONPs via warning labels and the potential influence of flavors on their appeal in diverse populations, including youth.

For nicotine concentration, the study findings showed that adults perceived ONPs displaying 6 mg nicotine concentration on the package to be less harmful and less addictive compared with ONP packaging that did not display the nicotine concentration. They also perceived ONPs displaying 3 mg nicotine concentration on the package to be less substitutable for cigarettes than ONPs that did not display the nicotine concentration on the package. ONPs are available with nicotine concentrations ranging from 2 mg to >10 mg per portioned pouch; this information is virtually universal on ONP packaging [24], and it is prominently emphasized in ONP advertising [26]. Low perceived risks are consistently associated with tobacco use behavior [49,50], suggesting this feature of ONP packaging may promote ONP use by influencing perceived risks. For other tobacco products, quantitative information about constituents (including nicotine) on packaging and advertising has been shown to mislead consumers about the potential risks, and in some settings it is prohibited on packaging and advertising [51]. To our knowledge, this study provides some of the first published evidence on how nicotine concentration on ONP packaging can affect consumer perceptions. Although these explanations are speculative, it is possible that consumers perceive higher nicotine concentration ONPs would translate to less frequent use and thus lower risks of health harm and addiction. It is also possible that consumers draw broad judgements about how ONP nicotine concentration affects nicotine delivery relative to other tobacco products, such as the observed pattern that 3 mg (but not 6 mg) ONP packages were viewed as less substitutable for cigarettes. 

Given the wide range of available ONP nicotine concentrations and the emphasis on nicotine concentration in ONP packaging and advertising, our findings highlight the importance of further research to understand how information about nicotine on ONP packaging affects consumer perceptions and ONP use behavior. There are other aspects of ONP packaging and advertising that we did not examine that are important topics of future study as well. For example, many ONP manufactures now claim to use synthetic nicotine, and such products use “tobacco free” and “synthetic” claims on the packaging and marketing [6]. In future studies, it will be important to examine how “tobacco free” and “synthetic” claims on ONP packaging impact consumer perceptions, and whether they affect the impact of other packaging features (e.g., nicotine concentration and addiction warnings). This evidence can inform potential regulations of ONP packaging and pre-market review of new tobacco products by the U.S. FDA [19,21] and similar regulatory agencies in other settings. 

These findings should be interpreted considering limitations of the study. We conducted the study with a convenience sample of adult tobacco users and non-users recruited from a single geographic area. Although for experimental tobacco research studies, convenience samples provide consistent results with population-based samples [52], this could have impacted our findings, such as the higher prevalence of awareness and use of ONPs in the sample relative to other published data [8]. This also limits the potential generalizability of the findings to broader populations. Among tobacco users, we focused on adults who smoked cigarettes and used ST. In future studies, it will be important to examine how ONP packaging characteristics affect perceptions and use behavior in adults who use other tobacco and nicotine products, such as electronic cigarettes. We did not include youth in our study, and research to examine the appeal of ONPs to youth is important. Our experimental design was informed by the published evidence on the popular ONPs at the time of the study (e.g., brand and flavor); however, our findings are limited to a single ONP brand and a limited range of flavors and other factors. We used pack colors that aligned with flavors consistent with the ONP manufacturers’ practices to maintain external validity in our design, but due to this we cannot disentangle the effects of flavor and pack color. Future studies can build from our results by testing other ONP brands, a wider range of product characteristics, and by independently examining the effects of packaging features such as color and flavor. We focused on measures of product perceptions, and research on how packaging and marketing affects ONP trial and use is needed to understand the potential public health effects.

## 5. Conclusions

The study provided some of the first experimental evidence on the effects of ONP packaging features on adult tobacco users’ and non-users’ product perceptions. The results highlighted that consumers’ perceptions of ONPs may be influenced by nicotine concentration displayed on ONP packaging. As the number of ONP brands proliferate, marketing increases, and ONP popularity grows, continued research to understand how features of ONP packaging and other forms of marketing emphasizing nicotine (e.g., the nicotine concentration and “tobacco free” nicotine claims) impact consumers will be important to capture their potential public health effects.

## Figures and Tables

**Table 1:** Sample characteristics (n = 301).

	% (n)	M (SD)
Age		35.7 (11.9)
Sex		
Male	77.1% (232)	
Female	22.9% (69)	
Race		
Black/African American	4.3% (13)	
White	92.0% (277)	
Other Non-White	3.7% (11)	
Ethnicity		
Hispanic	2.3% (7)	
Non-Hispanic	97.7% (294)	
Education		
College graduate or beyond	46.8% (141)	
Some college or less	53.2% (160)	
Income		
>$50,000	30.2% (91)	
≤$50,000 or Prefer not to say	69.8% (210)	
Subjective Financial Situation		
Poor	8.6% (26)	
It varies	2.7% (8)	
About average	60.5% (182)	
Pretty well off	28.2% (85)	
Employment		
Employed Full or Part Time	77.4% (233)	
Unemployed	7.6% (23)	
Disabled or Retired	8.3% (25)	
Student	6.6% (20)	
Tobacco Use Status		
Exclusive Cigarette Smoker	17.6% (53)	
Exclusive ST User	40.2% (121)	
Dual User of Cigarettes, ST	16.3% (49)	
Non-tobacco User	25.9% (78)	
Past 30 Day Use Of:		
Large Cigar	11.3% (34)	
Little Cigar	9.3% (28)	
Cigarillo	7.6% (23)	
Electronic Cigarette	18.6% (56)	
Waterpipe	3.3% (10)	
ONP Awareness		
Aware	68.1% (205)	
Not aware	31.9% (96)	
Ever Used ONPs		
Yes	41.7% (125)	
No	58.3% (175)	
Concentration Condition		
None displayed	33.2% (100)	
3 mg	35.9% (108)	
6 mg	30.9% (93)	
Flavor Condition		
Coffee	24.6% (74)	
Dark Frost	26.2% (79)	
Mint	23.9% (72)	
Smooth	25.2% (76)	
Warning Label Condition		
Present	50.2% (151)	
Absent	49.8% (150)	

ST = smokeless tobacco; ONP = Oral Nicotine Pouch.

**Table 2:** Overall Means for Perceptions of Oral Nicotine Pouches and Linear Regression Results.

	Perceived Substitutability for Cigarettes	Perceived Substitutability for ST	Perceived Harm	Perceived Addictiveness	Risk Appraisals—Harm	Risk Appraisals—Addiction
Overall Mean (SD)	5.0 (1.7)	5.8 (1.5)	2.6 (0.8)	3.2 (0.8)	3.7 (1.5)	4.5 (1.5)
Linear Regression Results *β* (95% CI)						
User Status						
Cigarette Smoker	0.32 (−0.29, 0.93)	0.08 (−0.46, 0.62)	**−0.53** **(−0.79, −0.28)**	**−0.43** **(−0.72, −0.15)**	**−1.03** **(−1.50, −1.09)**	**−0.98** **(−1.50, −0.36)**
ST User	0.18 (−0.31, 0.68)	0.22 (−0.22, 0.66)	**−0.95** **(−1.15, −0.74)**	**−0.22** **(−0.46, 0.01)**	**−1.48** **(−1.86, −1.09)**	**−0.77** **(−1.20, −0.36)**
Dual User	0.56 (−0.06, 1.18)	0.33 (−0.22, 0.88)	**−0.86** **(−1.12, −0.60)**	**−0.50** **(−0.79, −0.21)**	**−1.46** **(−1.93, −0.98)**	**−1.13** **(−1.65, −0.60)**
Non-user	Ref	Ref	Ref	Ref	Ref	Ref
Warning Label						
Yes	0.32 (−0.07, 0.71)	0.33 (−0.21, 0.67)	0.08 (−0.09, 0.24)	0.17 (−0.01, 0.36)	−0.03 (−0.34, 0.27)	0.25 (−0.08, 0.59)
No	Ref	Ref	Ref	Ref	Ref	Ref
Nicotine Concentration						
3 mg	**−0.49** **(−0.96, −0.02)**	−0.11 (−0.52, 0.31)	0.00 (−0.20, 0.20)	−0.09 (−0.31, 0.13)	0.00 (−0.36, 0.35)	−0.11 (−0.51, 0.29)
6 mg	−0.23 (−0.72, 0.26)	0.27 (−0.17, 0.70)	**−0.23** **(−0.44, −0.02)**	**−0.28** **(−0.51, −0.05)**	**−0.50** **(−0.88, −0.12)**	**−0.53** **(−0.95, −0.11)**
None Displayed	Ref	Ref	Ref	Ref	Ref	Ref
Flavor						
Cool Mint	−0.04 (−0.60, 0.52)	0.00 (−0.50, 0.50)	0.07 (−0.16, 0.31)	−0.18 (−0.44, 0.08)	0.17 (−0.26, 0.61)	−0.33 (−0.81, 0.14)
Dark Frost	−0.35 (−0.90, 0.19)	−0.32 (−0.80, 0.17)	0.11 (−0.13, 0.34)	−0.13 (−0.39, 0.12)	0.22 (−0.20, 0.64)	−0.40 (−0.86, 0.07)
Smooth	−0.50 (−1.05, 0.06)	0.18 (−0.32, 0.67)	0.05 (−0.18, 0.29)	−0.11 (−0.37, 0.15)	0.16 (−0.27, 0.58)	−0.09 (−0.56, 0.38)
Coffee	Ref	Ref	Ref	Ref	Ref	Ref

ST = Smokeless tobacco (chew, snuff, dip, and snus). Perceived substitutability for cigarettes/ST ranged from 1 to 7. Perceived harm/addictiveness ranged from 1 to 4. Risk appraisals for harm and addictiveness ranged from 1 to 7. The estimates in boldface font are statistically significant at *p* < 0.05.

## Data Availability

Data are available from the corresponding author upon request.
